# TdIF1: a putative oncogene in NSCLC tumor progression

**DOI:** 10.1038/s41392-018-0030-9

**Published:** 2018-10-19

**Authors:** Yujuan Zhang, Zhigang Wang, Yanqing Huang, Muying Ying, Yifan Wang, Juan Xiong, Qi Liu, Fan Cao, Rakesh Joshi, Yanling Liu, Derong Xu, Meng Zhang, Keng Yuan, Nanjin Zhou, James Koropatnick, Weiping Min

**Affiliations:** 1 Institute of Immunotherapy and College of Basic Medicine of Nanchang University, and Jiangxi Academy of Medical Sciences, Nanchang, China; 2 Jiangxi Provincial Key Laboratory of Immunotherapy, Nanchang, China; 3 Department of Environmental Health, Harvard T.H. Chan School of Public Health, Harvard University, Boston, USA; 4 Department of Surgery, Pathology and Oncology, University of Western Ontario, London, Canada; 5 Department of Preventive Medicine, School of Medicine, Shenzhen University, Shenzhen, China; 6 Institute of Translational Medicine, Nanchang University, Nanchang, China

## Abstract

TdT-interacting factor 1 (TdIF1) is a ubiquitously expressed DNA- and protein-binding protein that directly binds to terminal deoxynucleotidyl transferase (TdT) polymerase. Little is known about the functional role of TdIF1 in cancer cellular signaling, nor has it previously been identified as aberrant in any type of cancer. We report here for the first time that TdIF1 is abundantly expressed in clinical lung cancer patients and that high expression of TdIF1 is associated with poor patient prognosis. We further established that TdIF1 is highly expressed in human non-small cell lung cancer (NSCLC) cell lines compared to a normal lung cell line. shRNA-mediated gene silencing of TdIF1 resulted in the suppression of proliferation and anchorage-independent colony formation of the A549 adenocarcinoma cell line. Moreover, when these TdIF1-silenced cells were used to establish a mouse xenograft model of human NSCLC, tumor size was greatly reduced. These data suggest that TdIF1 is a potent regulator of lung tumor development. Several cell cycle-related and tumor growth signaling pathways, including the p53 and HDAC1/2 pathways, were identified as participating in the TdIF1 signaling network by in silico analysis. Microarray, transcriptome and protein-level analyses validated p53 and HDAC1/2 modulation upon TdIF1 downregulation in an NSCLC cellular model. Moreover, several other cell cycle regulators were affected at the transcript level by TdIF1 silencing, including an increase in CDKN1A/p21 transcripts. Taken together, these results indicate that TdIF1 is a *bona fide* tumor-promoting factor in NSCLC and a potential target for therapy.

## Immune protein involved in lung cancer

A protein involved in the immune system also plays a role in the most common type of lung cancer. Weiping Min, of the University of Western Ontario in Canada, and international colleagues found, for the first time, that the protein TdIF1 is significantly upregulated in non-small cell lung cancer (NSCLC) tissues in patients. High expression levels of this protein were correlated with poor prognosis. NSCLC tumor tissues grown in mice where TdIF1 expression was ‘knocked down’ were significantly smaller than in those without TdIF1 knockdown. Further analyses showed the protein was involved in known cell signaling pathways with roles in NSCLC progression. The findings indicate TdIF1 should be further investigated as a biomarker of NSCLC or as a molecular target for its treatment.

## Introduction

Lung cancer is the most common cancer globally, after basal skin cancer, and the deadliest of human cancers. This is primarily due to its rapid progression into metastatic stage IV before detection, especially the non-small cell lung cancer (NSCLC) subtype.^1^ NSCLC accounts for 80–95% of all lung cancer prognoses.^2^ Current lung cancer treatments also face numerous challenges, including the complexity and diversity of lung cancer subtypes and a penchant for acquired resistance to therapy.^3^ This highlights the importance of identifying novel regulatory molecules in lung cancer progression to develop effective diagnostics and targeted therapy. The lack of targetable mutations in 50% of NSCLC also underscores the importance of the identification and validation of drugable targets.^4^ The identification of novel regulatory molecules in lung cancer progression (biomarkers and/or therapeutic targets) is therefore clinically relevant, allowing for novel diagnostics to be developed for early detection and for effective individual or combined targeted therapies. This strategy forms the foundation of personalized medicine, especially in the therapy of resistant subtypes of cancer.^5,6^ A first step is therefore to identify and validate novel molecules, or putative oncogenes, to investigate translational and clinical methodologies.^7^

Terminal deoxynucleotidyl transferase-interacting factor 1 (TdIF1) is a ubiquitously expressed DNA-binding protein that is homologous to the transcription factor p65/NF-κB.^8^ However, it has an unknown role in cancer progression. The two isoforms of TdIF (*TdIF1/2*) are regulators of terminal deoxynucleotidyl transferase (TdT), which is a template-independent DNA polymerase involved in V(D)J recombination.^9^ Although TdIF1 is a sparsely studied transcription factor in the cancer context, it was identified as a component of the MiDAC (mitotic deacetylase) epigenetic complex, composed of heteromultimers of TdIF1, Trep-132/TRERF1 and HDAC1/2.^10^ Moreover, it has been shown that HDAC1/2 small molecule inhibitors that are already in clinical trials target TdIF1 directly, along with HDAC1/2, to inhibit the progression of cancers, including NSCLC.^11^ More importantly, HDAC1/2 has been implicated in the regulation of the omnipotent cell cycle and apoptosis transcription factor p53/TP53 and in CDKN1A/p21 regulation.^12^ The p53/TP53 tumor suppressor takes on enhanced oncogenic functions when mutated in nearly half of all cancers studied.^13^ However, less is known about the functional role of TdIF1 in HDAC1/2-p53 cellular signaling in cancer. Its significance in aggressive cancers, such as NSCLC tumor growth and metastasis, remains unknown. Therefore, the potential of TdIF1 as a biomarker or molecular target for lung cancer therapy remains unexplored.

In this study, we report for the first time that TdIF1 is significantly upregulated in clinical NSCLC tissue samples. We confirmed that TdIF1 is abundantly expressed in tumors from clinical lung cancer patients compared to their adjacent nontumor tissues. We also confirmed the overexpression of TdIF1 in 3 human NSCLC cell lines compared to a normal lung cell line. We were able to knockdown TdIF1 transcription and subsequent translation using a short hairpin RNA (shRNA/shTdIF1) in A549 cells. Knockdown of TdIF1 resulted in the suppression of cell proliferation and anchorage-independent colony formation in A549 lung adenocarcinoma cells. More importantly, TdIF1 knockdown reduced tumor size in a nude mouse xenograft model of human NSCLC, which suggests that TdIF1 is also a potent regulator of NSCLC progression in vivo. A bioinformatics analysis, supported by microarray, transcription and protein expression profiling, suggested that the role of TdIF1 in cancer progression is associated with HDAC1/2-p53 mediated signaling. Moreover, there was a significant upregulation of CDKN1A/p21 transcript levels upon TdIF1 downregulation. This implicates TdIF1 in the transcription and epigenetic control of NSCLC progression.

## Materials and methods

### Animals

Nude mice (6-to 8-week-old males, 18–22 g) were purchased from Changsha Laboratory Animal Co., Ltd., and housed in an SPF (specific pathogen-free) grade animal center. The use of all mice in this study complied with the Regulations for the Administration of Affairs Concerning Experimental Animals of China and the ethics approval of the Institutional Animal Care and Use Committee of Nanchang University, China.

### Cell culture

The human lung adenocarcinoma cell lines A549, H1299, and H1975 and the normal human lung bronchial epithelial cell line BEAS-2B were obtained from the American Type Culture Collection (ATCC) and cultured in DMEM (Invitrogen Life Technologies, Carlsbad, CA, USA) with 10% FBS and standard amounts of l-glutamine, penicillin, and streptomycin at 37 °C in 5% CO_2_.

### Quantitative reverse transcription PCR (RT-q-PCR)

Total cellular or tissue RNA was isolated using Trizol (Trizol reagent, Invitrogen) and then used as a template for cDNA synthesis. q-PCR using gene-specific forward and reverse primers (Beijing Genomics Institution, Beijing, China) was performed in a Stratagene Mx 3000P QPCR System (Agilent Technologies, Lexington, MA) using SYBR Green PCR MasterMix (Life Technologies) according to the manufacturer’s protocol. The primers used for the amplification of human TdIF1 were 5′- ACTGAACGTGCGAGACAATGT-3′ (forward) and 5′-GCTCATGGGTCAATCTGGGTATT-3′ (reverse). Primers for human GAPDH were 5′-TGACTTCAACAGCGACACCCA-3′ (forward) and 5′-CACCCTGTTGCTGTAGCCAAA-3′ (reverse). Primers for human HDAC1 were 5′-CGCCCTCACAAAGCCAATG-3′ (forward) and 5′-CTGCTTGCTGTACTCCGACA-3′ (reverse); primers for human HDAC2 were: 5′-GCTTTGCCCTTCTACCA-3′ (forward) and 5′-ACTGAGGCACAGAGGTTAG-3′ (reverse); primers for human CDKN1A were: 5′-GGAAGACCATGTGGACCTGT-3′ (forward) and 5′-GGCGTTTGGAGTGGTAGAAA-3′ (reverse); primers for human CDK4 were: 5′-CAGATGGCACTTACACCCGT-3′ (forward) and 5′-CAACTGGTCGGCTTCAGAGT-3′ (reverse); primers for human Cyclin D1 were: 5′-CAGATCATCCGCAAACACGC-3′ (forward) and 5′-AAGTTGTTGGGGCTCCTCAG-3′ (reverse); primers for human CDK6 were: 5′-ACAGAGCACCCGAAGTCTTG-3′ (forward) and 5′-CTGGGAGTCCAATCACGTCC-3′ (reverse); primers for human CDC20 were: 5′-AATGTGTGGCCTAGTGCTCC-3′ (forward) and 5′-AGCACACATTCCAGATGCGA-3′ (reverse); and primers for human CDC25C were: 5′-GAACCCCAAAACGTTGCCTC-3′ (forward) and 5′-GTGGTAAGCTGAGTGGCAGT-3′ (reverse).

### Western blot

A549 cells were harvested and lysed, and the supernatant was collected and stored at −20 °C for future use. The protein concentration was determined by the Bio-Rad protein assay, and 40 µg of cell lysate from each group was separated on a 10% SDS-PAGE gel, transferred to a nitrocellulose membrane, and probed separately with mouse anti-human TdIF1, p53, Ac-p53, HDAC1, HDAC2 and GAPDH mAbs (Santa Cruz Biotechnology) according to the manufacturer's instructions, and the bands were visualized using an ECL assay kit (Pierce, Rockford, IL, USA).

### Immunohistochemistry (IHC)

The collected tumor tissues were fixed in 10% formalin and sectioned into 5-μm slices. The slices were stained with rat anti-TdIF1 antibody (1:50, Santa Cruz Biotechnology) overnight at 4 °C. A streptavidin-biotin-peroxidase complex with diaminobenzidine (DAB) was applied. The prepared specimen was examined under a microscope.

### Celigo image cytometry

A549 cells (3 × 10^5^ cells/well) were seeded in 96-well microplates and cultured for 5 days in the presence of calcein staining solution. The live cell numbers were counted daily using the Celigo image cytometer (Nexcelom Bioscience, Lawrence, MA, USA).

### Colony formation/soft agar assay

A total of 1000 cells/well of TdIF1-knockdown (KD) or WT A549 cells was seeded in triplicate in 6-well culture plates for 14 days, at which point the majority of single colonies contained greater than 50 cells. At the end of the experiment, the cells were fixed for 30–60 min with 4% paraformaldehyde and then stained with crystalline violet dye solution for 10 min. The images were taken with a digital camera, and colonies were counted.

### Human lung cancer xenograft model

The murine lung cancer model was established by subcutaneously inoculating 2 × 10^5^ TdIF1-KD or WT A549 cells. Tumor growth was monitored on alternate days. The tumor size was measured using calipers, and volumes were estimated using the following formula: tumor volume = 0.5 × width^2^ × length.

### Data analysis using The Cancer Genome Atlas (TCGA)

RNA-Seq data of 57 paired tissues (lung cancer tumor and adjacent normal tissue) were downloaded from the Cancer Genome Atlas Project (TCGA; https://tcgadata.nci.nih.gov/tcga/). Sequence reads were aligned using TopHat v 2.0.8 and quantified by Cufflinks 2.1.0. Differentially expressed genes (DEGs) were identified by Cuffdiff2.

### Gene microarray and pathway analysis

Gene expression profiles in TdIF1 knockdown and normal cells were analyzed by using an Affymetrix gene expression microarray. Total RNA from the human lung cancer cell line A549 with the TdIF1 gene stably knocked down and control A549 cells (*n* = 3 biological replicates) was isolated. RNA quality and the degree of degradation were assessed with the Agilent 2100 Bioanalyzer system using the RNA 6000 Nano LabChip® kit. A GeneChip Scanner 3000 was used to analyze the acquired microarray images. Quantile normalization and subsequent data processing were performed using the Affymetrix Microarray Suite 5.0 statistical algorithm. Differentially expressed genes were identified by filtering the criteria of the Benjamini-Hochberg-adjusted *P* value (<0.01) and fold change (≥2). IPA software (Ingenuity Pathway Analysis software tool) was used to investigate gene regulatory networks and functional relevance for differentially expressed genes. The Ingenuity Pathways Knowledge Base information is extracted from public databases and reviewed publications and is an extensive, manually curated database of functional interactions.^14^ A one-sided Fisher's exact test was performed to determine the significance of gene enrichment with a particular biologically relevant function.

### Tissue microarray construction

NSCLC tumors and normal lung tissues were obtained from the National Human Genetic Resources Sharing Service Platform (Shanghai, China). A tissue microarray containing 82 paired NSCLC cancerous and normal lung tissues was used to construct tissue microarray slides (Shanghai Biochip Company, Ltd., Shanghai, China). Briefly, holes with 0.6-mm diameters were made to preserve the tissues of selected tumor and adjacent tissues in paraffin blocks. Serial sections (0.66 μm) were cut from the arrayed paraffin block and placed onto glass slides. The tissue microarrays were validated by using HE and IHC staining. The histopathological categories of the non-small cell lung cancers were grade II–III. The IHC staining intensities (0–3) and the antibody positive rate values (0–3) of nuclear TdIF1 were standardized and interpreted as follows: intensity scores were 0 points (negative) or 1–3 (1+ to 3+); antibody positive rate scores were either 0 (negative staining) or scored as 1 (1–25% stained), 2 (26–50%), 3 (51–75%) or 4 (76–100%). The product of the intensity score and positive rate score was defined as the total score. Total scores were categorized into two groups based on a total score <4 as the antibody low expression group and ≥4 as the antibody high expression group.

### Statistics

Data are presented as the mean ± SD. Student's *t*-test (two-tailed) was applied to determine differences between two means. For the comparison of multiple groups, one-way ANOVA was used. Fisher’s exact test and the *X*^2^ test were used to compare the variables between groups. Kaplan–Meier analysis was used to compare the survival rates among groups. For all statistical analyses, differences with *P* values <0.05 were considered significant.

## Results

### TdIF1 is upregulated in lung cancer

**Fig. 1:**
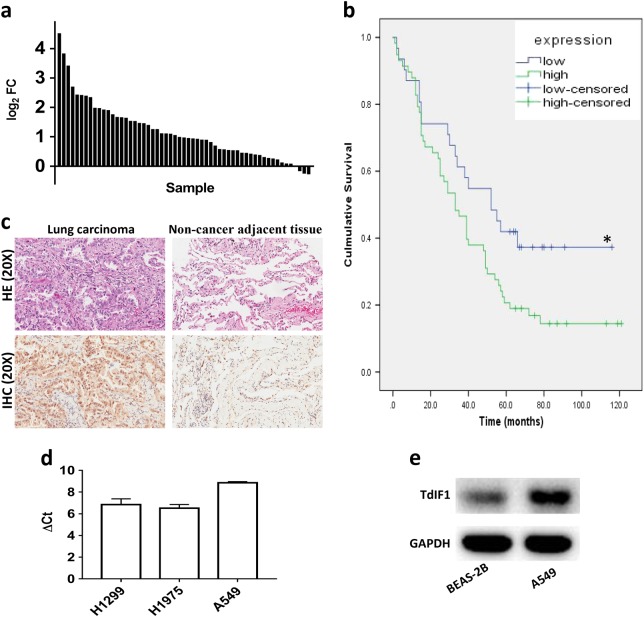
Expression of the TdIF1 gene in lung cancer. **a** The RNA-Seq data of 57 pairs of cancer and adjacent nontumor tissues from lung cancer patients were obtained from the TCGA common database and analyzed by Cuffdiff2. The fold changes in TdIF1 expression in lung cancer tissue vs noncancerous adjacent tissue were calculated and displayed. **b** Kaplan–Meier analysis of overall survival of patients with expression of TdIF1 in 82 lung adenocarcinoma patients (*P = 0.037). **c** H&E and immunohistochemical staining to detect TdIF1 expression in lung cancer and noncancer adjacent tissue. **d** Transcript abundance of TdIF1 in lung cancer cell lines. Expression of TdIF1 in 3 lung cancer cell lines. The three indicated lung cancer cell lines were cultured, and mRNA was collected. The expression of TdIF1 was determined by quantitative PCR. The abundance of TdIF1 expression was calculated in comparison to the internal control housekeeping gene, GAPDH. **e** Expression of TdIF1 in normal lung cell lines. Total protein (40 μg) was extracted from the normal human lung cell line BEAS-2B (bronchial epithelial cells) and the lung cancer cell line A549. Samples were subjected to western blotting using antibodies against TdIF1 and GAPDH. The data show one of three representative experiments. Error bars represent the standard deviation of three experiments

We first performed representational difference analysis with RNA-Seq information of 57 paired tissues (lung cancer tumor and adjacent normal tissue) from The Cancer Genome Atlas (TCGA) database. Significantly higher expression of TdIF1 was observed in NSCLC tumors than in adjacent lung noncancer tissues (Fig. 1a). We then examined the expression of TdIF1 in tumor tissues in NSCLC patients. The tissue microarray data showed that 54 of 82 lung cancers displayed high expression of TdIF1, whereas only 10 of 82 normal adjacent lung tissues showed an increase in TdIF1 (Table 1, *P* < 0.001). Further analysis revealed that TdIF1 was correlated with poor prognosis of lung cancer patients. Patients with low expression of TdIF1 demonstrated significantly higher survival rates than patients with high expression of TdIF1 in lung cancer tissue (Fig. 1b). Additionally, the expression of TdIF1 was associated with lymph node metastasis in non-small cell lung cancer patients. Increased expression of TdIF1 in patients was associated with tumor lymph node metastasis (TNM stage), with significant scores at the lymph node stage (N stage; Table 2; *P* = 0.024).

**Table 1:** Differential expression of nuclear TdIF1 in cancerous and normal lung tissues

	*n*	TdIF1 expression			
		High (%)	Low (%)	Chi-square value	*P* value
Lung carcinoma	82	54	28	49.610	*P* < 0.001
Normal lung tissues	82	10	72

**Table 2:** Correlation between TdIF1 expression and clinic pathological characteristics

	Variables	TdIF1 expression		Total	*Χ* ^2^	*P* value
		High	Low			
Age (year)					1.298	0.254
	≤60	24	17	41		
	>60	35	15	50		
Sex					1.298	0.254
	Female	24	17	41		
	Male	35	15	50		
TNM stage					3.613	0.057
	I/II	24	21	45		
	III/IV	30	11	41		
	null			5		
T stage					5.129	0.024*
	N0	20	18	38		
	N1/N2/N3	28	8	36		
	null			17		
M stage					0.558	0.455
	M0	57	32	89		
	M1	1	0	1		
	null					

Correlation analysis between TdIF1 expression and clinical indicators of lung adenocarcinoma patients was conducted using chi-square test and spearman analysis. *P* values <0.05 were considered statistically significant

*TNM stage* tumor lymph node and metastasis stage, *T* tumor, *N* lymph node, *M* metastasis

To further categorize the distribution of TdIF1 inside tumor cells, lung adenocarcinoma tissues and normal lung tissues were stained by H&E and anti-TdIF1 antibody (IHC). Lung adenocarcinoma showed nuclear atypia (Fig. 1c), while IHC showed positive staining of TdIF1 in both the nucleus and cytoplasm, although the expression in nuclei was more prominent (Fig. 1c). In contrast, the adjacent lung tissue showed the normal structure of the alveoli (Fig. 1c), and the expression of TdIF1 was significantly lower than what was observed in tumor tissue (Fig. 1c). Next, we detected the expression of TdIF1 in three human lung cancer cell lines, namely, A549 (alveolar epithelial, adenocarcinoma; NSCLC), H1299 (metastatic lymph node carcinoma; NSCLC), and H1975 (epithelial, adenocarcinoma, NSCLC). Our results indicated that among the three lung cancer cell lines, the highest expression of TdIF1 was in A549 cells, as measured by quantitative PCR (Fig. 1d). Furthermore, we confirmed that the expression of TdIF1 protein was significantly higher in A549 cells than in the normal human lung cell line BEAS-2B (bronchial epithelial cells), as detected by western blotting (Fig. 1e). Taken together, TdIF1 overexpression is associated with lung adenocarcinoma.

### TdIF1 is critical in tumor growth

To validate the role of TdIF1 in lung cancer, we constructed TdIF1-shRNA lentiviral vectors and assessed TdIF1 downregulation in A549 cells. To identify a specific sequence for TdIF1-shRNA, three siRNAs targeting TdIF1 were first used to investigate the efficacy of gene knockdown. All three TdIF1 siRNAs showed robust inhibitory effects (Supplementary Figure S1). The siRNA #1 sequence was used to construct the shRNA to further investigate the function of TdIF1 in lung adenocarcinoma in this study.

**Fig. 2:**
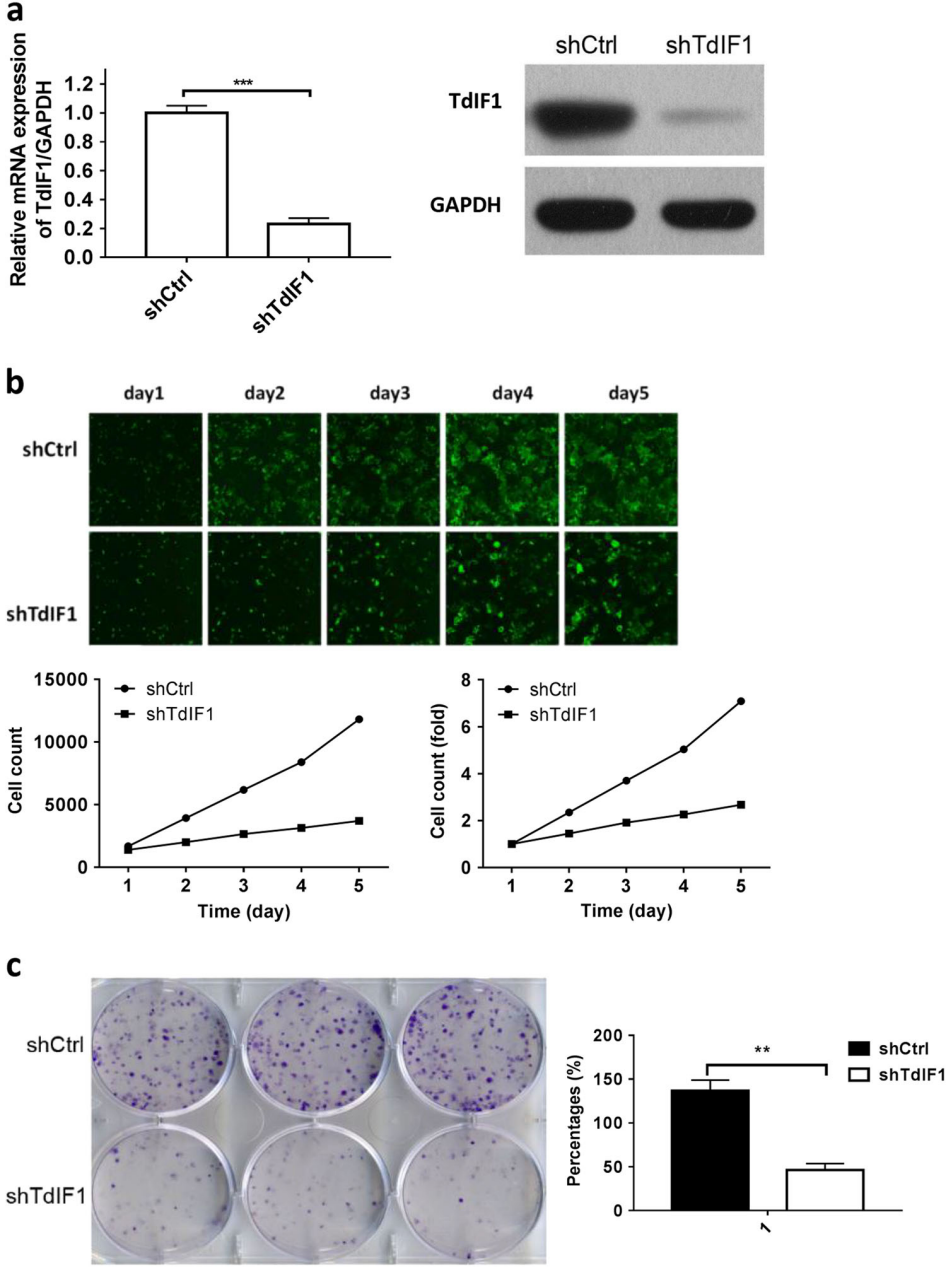
Suppression of tumor cell growth after shRNA-mediated gene knockdown of TdIF1 in A549 cells. **a** Knockdown of TdIF1 in A549 lung cancer cells via TdIF1-shRNA lentiviral vector. The lentiviral vector is a hU6-MCS-CMV-EGFP construct. The RNA sequence (TTCTCCGAACGTGTCACGT) was synthesized and ligated into the lentiviral vector. A549 cells were cultured and transfected with the TdIF1 shRNA lentiviral vector (shTdIF1) or a control nonspecific shRNA vector (shCtrl) at an MOI of 10 for 24 h. TdIF1 mRNA was detected by qPCR, and TdIF1 protein expression was detected by western blot. **b** In vitro suppression of cell proliferation by gene silencing of TdIF1. A549 cells were transfected with the lentiviral vector shTdIF1 or the control vector shCtrl at an MOI of 10 for 24 h. Live cells were stained with a green dye and detected by a Celigo image cytometer for 5 days. Cell images, cell number counts and cell proliferation fold changes are presented. **c** Decreased colony formation of A549 cells after gene knockdown of TdIF1. A549 cells were transfected with the lentiviral vector shTdIF1 or the control vector shCtrl at an MOI of 1:100 for 24 h. Approximately 1000 cells were plated in 6-well plates and cultured for 11 days. The cell clones were determined by staining with crystal violet. The crystal violet-stained colonies were imaged and counted (presented as a percentage) using a microscope. Error bars represent the standard deviation of three experiments (*P < 0.05; **P < 0.01)

Our data showed that the expression of TdIF1 was markedly knocked down by TdIF1-shRNA, which was confirmed by qPCR (Fig. 2a) and western blots (Fig. 2a). We used our established methods^15^ to generate stable TdIF1 knockdown (TdIF1-KD) lung carcinoma A549 cell lines using TdIF1-shRNA.

We then compared TdIF1-KD and wild type (WT) tumor cells by examining consequent biological phenotypic changes. The proliferation of TdIF1-KD cells was significantly suppressed compared to WT A549 cells, as observed using fluorescent microscopy (Fig. 2b). After gene knockdown of TdIF1, the number of proliferating cells was significantly reduced (Fig. 2b) by 8-fold (Fig. 2b). Moreover, TdIF1-KD cells formed significantly fewer colonies than WT lung carcinoma cells (Fig. 2c), which further confirms that TdIF1 is a tumor-promoting factor in lung adenocarcinoma in vitro.

### TdIF1 is a potential target for the suppression of NSCLC in vivo

**Fig. 3:**
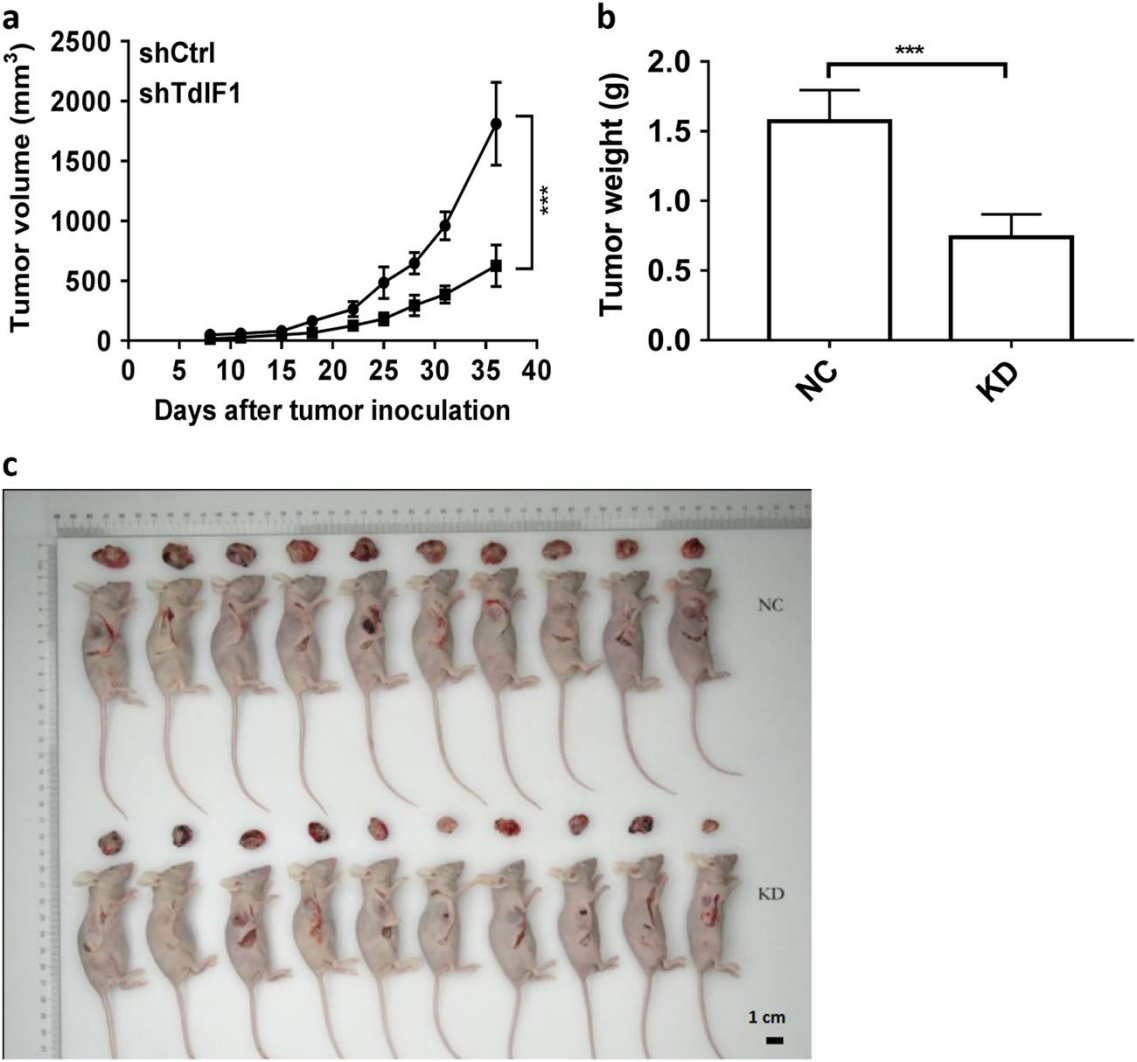
Effect of TdIF1 knockdown on tumor profiles in a xenograft NSCLC model established in nude mice. **a** Suppression of tumor cell growth after gene knockdown of TdIF1 in A549 cells. A549 cells were transfected with shTdIF1 (KD) and control shCtrl (NC) vectors. Twenty-four hours after gene transfection, 5 × 10^5^ cells were inoculated into immune-deficient nude mice. Tumor growth was measured on the indicated days and was estimated using the following formula: tumor volume = 0.5 × length × width.^2^ **b** Tumor weights were measured at the endpoint of experiments. Tumor weight was decreased between WT (NC) and TdIF1-KD mice. **c** Tumor size. The tumor size at the end of the experiments was photographed along with the animals, indicating a distinct decrease in tumor size with no significant differences in endpoint animal size. Error bars represent the standard deviation of 10 experimental or control mice (***P < 0.001)

We next investigated the in vivo role of TdIF1 in NSCLC using a human tumor xenograft model that we established in nude mice. After inoculation of TdIF1-KD and control TdIF1-WT A549 cells into immune-deficient nude mice, tumor growth and progression were monitored by measuring tumor size. While tumors grew aggressively in mice inoculated with WT A549 cells, tumor growth was significantly suppressed in mice injected with TdIF1-KD lung adenocarcinoma cells (Fig. 3a). Additionally, the tumor weight (Fig. 3b) and size (Fig. 3c) were significantly reduced after antisense knockdown of TdIF1 in lung cancer cells at the endpoint of the study, suggesting that TdIF1 is critical for tumor growth in vivo and is a putative molecular target.

### TdIF1 regulates the HDAC-p53 axis

**Fig. 4:**
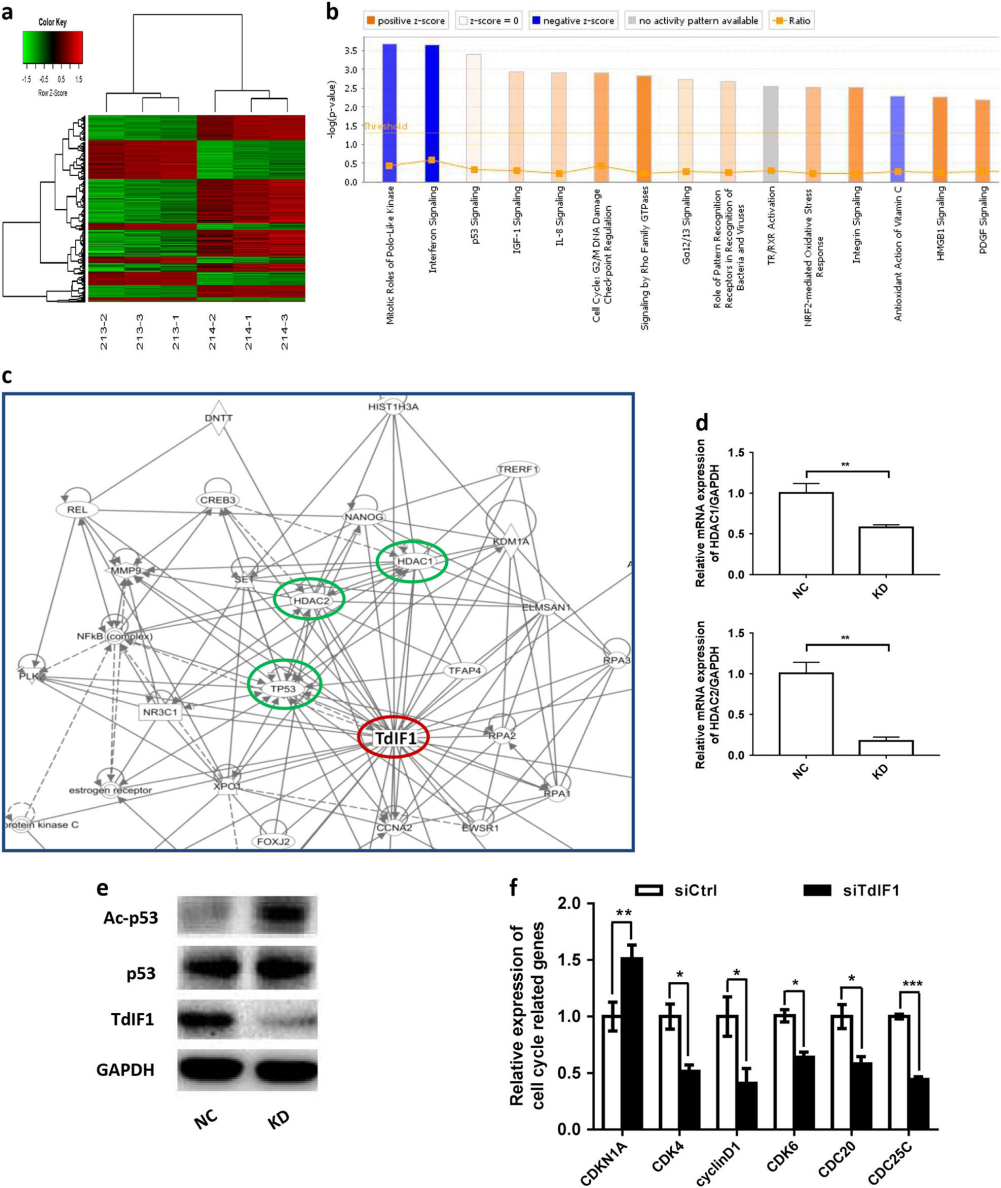
TdIF1 gene interaction pathway. **a** Hierarchical clustering of differential gene expression in A549 cells transfected with shTdIF1 (KD samples 213–2, 213–3 and 213–1) vs. control shCtrl (NC: 214–2, 214–1, and 214–3) vector. In the heat map, columns represent samples, and rows represent genes. The upper dendritic structure is the aggregation or classification of all samples according to the expression profile of different genes, while the left dendritic structure indicates the expression pattern aggregation of different genes. Red represents relatively high gene expression, green represents relatively low gene expression, black indicates no significant change in gene expression, and gray indicates undetected genes. Fold change >1.5 and FDR<0.05 were used as standards for screening. **b** The top 15 significant pathways of differential gene expression in A549 cells transfected with shTdIF1 vs. control shCtrl vector using Pathway Enrichment Analysis. The *X*-axis is the name of the pathway, and the *Y*-axis is the significance level of enrichment (negative logarithmic transformation at the base of 10). Among these, orange represents activated pathways (*Z*-score > 0), blue represents suppressed pathways (*Z*-score < 0), and the gradation of orange and blue represents the degree of activation or suppression (in accordance with the internal algorithm and IPA standard that a *Z*-score > 2 for a pathway represents significantly activated, while a *Z*-score < -2 for a pathway represents significantly suppressed); the ratio represents the number of differentially expressed genes in this signaling pathway to the number of all genes contained in this pathway. **c** In silico analysis of the TdIF1 gene interaction network. The protein interaction network map was built using the Ingenuity Pathway Analysis software (Qiagen) to determine the putative interactions of interest (blue circles) with TdIF1 (red circle). **d** Gene expression after gene silencing of TdIF1. The lung cancer cell line A549 was cultured and transfected with TdIF1 shRNA lentiviral vector (KD) or control nonspecific siRNA vector (NC) at an MOI of 10 for 24 h. Total RNA was collected from the cells after gene silencing, and the expression of HDAC1 and HDAC2 was detected by qPCR. **e** p53 and acetyl-p53 expression levels in TdIF1 knockdown A549 cells. A549 cells were transfected with siTdIF1 (KD) or control nonspecific siRNA vector (NC) for 72 h, followed by immunoblotting analysis using the indicated antibodies. **f** Expression of cell cycle-related genes after gene silencing of TdIF1. The A549 cells were transfected with siTdIF1 or control nonspecific siRNA vector (NC) for 48 h. The expression of CDK4, cyclin D1, CDK6, CDC20 and CDC25C was detected by quantitative RT–PCR. Error bars represent the standard deviation of three experiments (*P < 0.05)

We demonstrated that TdIF1 is significantly upregulated in lung cancer tissues (Fig. 1) and is an essential factor for tumor growth in vitro (Fig. 2) and in vivo (Fig. 3). However, the signaling pathways of TdIF1 in lung cancer progression are unknown. We thus compared the differential expression of genes between TdIF1-KD and WT lung adenocarcinoma using a microarray (Fig. 4a). Pathway enrichment analysis demonstrated that multiple cell proliferation and survival signaling pathways, such as the p53/TP53 pathway, were associated with TdIF1 (Fig. 4b).

In addition, bioinformatics analysis and the in vitro DNA selection assay (SELEX) have shown that TdIF1 may regulate over 300 genes, including cancer-linked genes such as RAB20, by binding to a 5′-GNTGCATG-3′ consensus motif in the 3′UTR following an AT-rich tract.^16^ However, most of these gene interactions have not been investigated. Therefore, we conducted an Ingenuity Pathway Analysis (IPA) to identify the molecules regulated by TdIF1. Several tumor growth and cell cycle-related signaling pathways, including the p53, HDAC1/2 and TReP132/TRERF1 pathways, were highlighted as potential interactors of the TdIF1 signaling network (Fig. 4c).

TdIF1 is also believed to function in transcription regulation via an epigenetic mechanism because it binds directly to histone deacetylases HDAC1/2 in a MiDAC complex.^17^ It has also been reported that HDAC1/2 directly suppresses p53 pathway activity by deacetylating lysine residues on the C-terminus of p53.^18^ Therefore, to confirm the IPA prediction of TdIF1-associated HDAC1/2 expression, we detected alterations of HDAC1/2 in TdIF1-modulated A549 cells. The results indicated that knockdown of TdIF1 resulted in the downregulation of HDAC1 by 41.6% (Fig. 4d) and of HDAC2 by 82.5% (Fig. 4d). We also found evidence that the knockdown of TdIF1 resulted in an upregulation of acetylated-p53 (Ac-p53) levels in TdIF1-modulated A549 cells compared with nonsilenced control cells (Fig. 4e). In addition, our qPCR data showed that TdIF1 could regulate the expression of cell cycle-related genes, including CDKN1A/p21, CDK4, cyclin D1, CDK6, CDC20 and CDC25C. Notably, the cyclin-dependent kinase inhibitor CDKN1A/p21Cip1 was upregulated while CDKs (CDK4 and CDK6), cyclin D1, CDC20, and CDC25C were downregulated in siTdIF1 A549 cells (Fig. 4f). These results indicated that the HDAC-p53 axis, along with the CDKN1A/p21 effector, may be critical components of downstream TdIF1 signaling.

## Discussion

There are many challenges in the targeted treatment of lung cancer, including rapid metastasis before detection and the complexity, diversity and acquired resistance to therapy in cancer subtypes. There are no validated targets for more than 50% of NSCLC etiologies, which make up 80–95% of all lung cancer cases.^4^ Moreover, traditional treatments for NSCLC have reached what is referred to as a “therapeutic ceiling”, including limitations of surgery, radiotherapy, chemotherapy and combinations of radio- and chemotherapy. This is especially evident with regard to resistant cancer subtypes. Although surgery is an effective treatment for NSCLC, the majority of patients are diagnosed at a late metastatic state. Therefore, lung cancer treatment usually involves systemic chemotherapy combined with radiotherapy, but the therapy is debilitating, with limited effects on the patient. In recent years, EGFR tyrosine kinase gene mutations, as well as the EML4-ALK gene fusion, were determined to be a cause of NSCLC.^19^ This has led to the development of a variety of molecular-targeted drugs in preclinical and clinical studies. Although specific patient populations respond well to treatment with these new therapies, most patients still do not respond to them, even in combinatorial therapy modes. Therefore, new molecular-targeted therapies with specific characteristics are necessary for the treatment of advanced NSCLC.

In addition, therapies based on immune inhibitory molecule blockade, such as programmed cell death receptor 1 (PD1) in T cells, can effectively promote antitumor immune responses.^20^ However, in patients treated with a representative PD1 inhibitor Nivolumab, the 1-year progression-free survival was only 19%, which therefore showed no advantage over traditional chemotherapy drugs.^21^ This sparse therapeutic landscape makes it imperative to identify and validate new molecular targets in NSCLC progression. Therefore, the search for new target molecules, including the transcriptional regulators and epigenetic modulators of tumor growth factors, has important clinical significance.^22^ In this study, we identified the overexpression of TdIF1 in NSCLC patient digital data by data mining of the TCGA database and validated this analysis in patient-derived tumor and adjacent normal tissue samples. TdIF1 gene silencing in proof-of-concept in vitro and in vivo experiments inhibited proliferation and anchorage-independent growth of A549 lung cancer cells. To study the role of TdIF1 in NSCLC in vitro and in vivo, we also established a TdIF1 knockdown (TdIF1-KD) stable lung cancer cell line and a human NSCLC tumor xenograft model. Our initial studies indicate that downregulation of TdIF1 gene expression can inhibit tumor growth, suggesting that TdIF1 may be a new target for lung cancer-targeted therapy.

TdT interacting factor 1 (TdIF1) was first identified as a protein that directly binds to terminal deoxynucleotidyl transferase (TdT) polymerase.^23^ TdT polymerase has been investigated in the context of how it contributes to the diversity of immunoglobulins and T-cell receptors in lymphocytes.^24^ TdIF1 regulates TdT activity^8,23^ and controls TdT degradation through the BPOZ-2-mediated ubiquitin proteasome system.^25,26^ These studies also highlight that TdIF1 regulates TdT in lymphocytes at the post-translational level. However, TdIF1 can also bind DNA and, moreover, is expressed ubiquitously, suggesting that TdIF1 possesses additional biological functions in non-lymphoid tissues. This study demonstrated for the first time that TdIF1 is highly expressed in tumor cells.

TdIF1 has recently been found to be a DNA- and protein-binding protein and is homologous to the p65/NF-κB transcription factor. It binds DNA as a dimer, and its TdT-regulating, dimerization and nuclear localization characteristics have been elucidated.^27^ TdIF1 has been identified as a putative transcription factor for multiple oncogenes, including RAB20.^28^ TdIF1 may also transcriptionally regulate over 300 genes that have not been investigated in a cancer context.^29^ Bioinformatics analysis and in vitro DNA selection assays (SELEX) indicate that TdIF1 binds a 5′-GNTGCATG-3′ consensus motif in the 3′UTR following an AT-rich tract found in these gene promoters.^16^ However, these studies were performed in fibroblasts, and whether the 300 putative gene promoter targets of TdIF1 have relevance in cancer, especially in NSCLC, has not been investigated.^29^ In this study, we identified for the first time that TdIF1 is highly expressed in NSCLC patients. We found that TdIF1 is highly upregulated in clinical lung cancer tissues, which is associated with the prognosis of lung adenocarcinoma (Fig. 1). We confirmed that TdIF1 is abundantly expressed in three human NSCLC cell lines, namely, A549, H1299 and H1975. shRNA-mediated downregulation of TdIF1 in A549 cells inhibited cell proliferation and colony formation (Fig. 2). Moreover, TdIF1 knockdown reduced tumor size in a mouse xenograft model of human NSCLC (Fig. 3). These data suggest that TdIF1 is a potent regulator of lung cancer. However, its role in established cancer signaling pathways, such as those involving p53 and HDAC1/2, has not been explored.

The transcription factor p53/TP53, which is a tumor suppressor that turns aggressively oncogenic when mutated, is dysfunctional in more than half of all cancers studied.^30^ This is considered a cancer hallmark, including in NSCLC.^31,32^ p53 is a master transcription factor and functions by directing cell cycle arrest or inducing apoptosis in response to cellular stress, including DNA damage and oncogenic stimulation.^33^ p53 directly regulates cell cycle and apoptosis genes, such as cyclin G, bax, and CDKN1A/p21^*WAF1/CIP1*^.^34^ p53 can be regulated at the gene or protein level by various modifications, including acetylation.^35^ Epigenetic or post-translational reprogramming, including histone and non-histone acetylation, can change gene expression patterns or p53 protein activity. Acetylation profiles, including those of p53, are distinct in lung cancer progression, including in NSCLC.^36^ We demonstrated in this study that TdIF1 is associated with the p53 pathway.

The acetylation of p53, including on K120/373/381/K382, enhances anticancer activity.^37^ The reversible regulation of p53, via the deacetylation of the C-terminal regulatory region, is primarily regulated by histone deacetylases (HDACs), including HDAC1/2. HDACs remove acetyl groups from histone and non-histone lysines, either restricting DNA accessibility and transcription or modifying protein activity post-translationally.^38^ HDACs can therefore regulate normal and cancer-associated gene expression.^39^ HDACs are known to modulate multiple processes in chromatin organization, including repressing the transcription of genes mediating apoptosis and the expression of cell cycle regulators such as cyclins and CDKs.^40^ However, they can also directly target lysine residues on protein substrates for deacetylation and downregulate protein activity, including p53.^18^ Moreover, HDAC1/2 have been identified to be overexpressed in certain cancers, and small molecule HDAC inhibitors (HDACi) are being investigated as lung cancer chemotherapeutics.^41^ Epigenome-modulating lung cancer drugs are already in clinical research and in drug development pipelines.^42,43^ Therefore, HDAC1 inhibition is a potent strategy to prevent NSCLC progression.^4^ Interestingly, TdIF1 has been implicated as a component of the MiDAC complex, through which HDAC1/2 are functional, especially in a cancer context.^17^ Moreover, HDAC inhibitors can directly target TdIF1.^17^ To identify interacting molecules of TdIF1 in modulating lung cancer growth, such as p53 and HDAC1/2, we investigated the differential gene expression between TdIF1-KD and WT lung cancers. Through our microarray study, we identified that p53 expression and the activity of the p53 pathway were altered after TdIF1 was downregulated. Additional analysis using the Ingenuity Pathway methodology further identified several molecules in the TdIF1 signaling pathway, including p53, HDAC1/2 and TReP132/TRERF1, the latter two being components of the MiDAC complex (Fig. 4c). In addition, we demonstrated that knockdown of TdIF1 resulted in activation of the p53 pathway (Fig. 4b) and lowered transcript expression of HDAC1 (Fig. 4d) and HDAC2 (Fig. 4d). Recent studies have reported that HDACs are associated with the deacetylation of p53,^44–56^ while acetylated p53 upregulates CDKN1A/p21^WAF1/CIP1^ to direct cell cycle arrest at G1 phase.^57,58^ To ascertain the relationship between TdIF1 and p53, we investigated p53 expression levels and acetyl-p53 levels in A549 cells treated with siTdIF1 and control nonspecific siRNA. We found that knockdown of TdIF1 resulted in upregulation of acetyl-p53 levels in A549 cells compared with control cells (Fig. 4e). Notably, our data also showed that the cyclin-dependent kinase inhibitor CDKN1A/p21 was upregulated while CDKs (CDK4 and CDK6), cyclin D1, CDC20 and CDC25C were downregulated in siTdIF1 A549 cells (Fig. 4f). These results highlight that the TdIF1-HDAC-p53 axis is involved in regulating A549 cell proliferation, which may be a prominent mechanism for NSCLC progression.

In conclusion, we reveal for the first time that TdIF1 is significantly upregulated in clinical non-small cell lung cancer (NSCLC) tissues of patients. We further confirmed that TdIF1 is involved in cell proliferation and anchorage-independent growth in an adenocarcinoma cell line. More importantly, TdIF1 downregulation in an NSCLC xenograft model suppressed lung tumor growth. Additionally, we provide data from bioinformatics, microarray and transcriptome and protein-level analyses that support our hypothesis that the HDAC1/2-p53 signaling pathway is involved in TdIF1 signaling in NSCLC cancer progression. This proof-of-concept study provides a platform for developing TdIF1 as a novel biomarker or molecular target for NSCLC therapy in preclinical and clinical investigations. TdIF1 may also prove to be a *bona fide* oncogene that requires investigation in other solid, aggressively metastatic cancers.

## Electronic supplementary material

Supplemental Information
