# Supplementary material for: TdIF1: a putative oncogene in NSCLC tumor progression
Source: Signal Transduct Target Ther. 2018 Oct 19;3:28. doi: 10.1038/s41392-018-0030-9 (PMC6194072; doi:10.1038/s41392-018-0030-9)
Supplement: Supplementary file 1 — Supplemental Information [file 41392_2018_30_MOESM1_ESM.docx]

**Supplementary Material**

**TdIF1: A putative oncogene in NSCLC tumor progression**

Yujuan Zhang^1,2,6,*,#^, Zhigang Wang^1,2,*^, Yanqing Huang^1,2*^, Muying Ying^1,2^, Yifan Wang^1,2,5^, Juan Xiong^3^, Qi Liu^1,2^, Fan Cao^1,2^, Rakesh Joshi^5^, Yanling Liu^1,2^, Derong Xu^4^, Meng Zhang^1,2,5^, Keng Yuan^1,2^, Nanjin Zhou^1,2^, James Koropatnick^5^, Weiping Min^1,2,^^5,#^

1. Institute of Immunotherapy and College of Basic Medicine of Nanchang University, and Jiangxi Academy of Medical Sciences, Nanchang, China;

2. Jiangxi Provincial Key Laboratory of Immunotherapy, Nanchang, China;

3. Department of Preventive Medicine, School of Medicine, Shenzhen University, Shenzhen, China;

4. Institute of Translational Medicine, Nanchang University, Nanchang, China;

5. Department of Surgery, Pathology and Oncology, University of Western Ontario, London, Canada;

6. Department of Environmental Health, Harvard T.H. Chan School of Public Health, Harvard University, Boston, USA.

**^*^** These authors contributed equally to this work.

**^#^ Correspondence to**: Dr. Yujuan Zhang (email: yujuanzhang@ncu.edu.cn) or Dr. Weiping Min (email: weiping.min@uwo.ca).

**Figure S1**


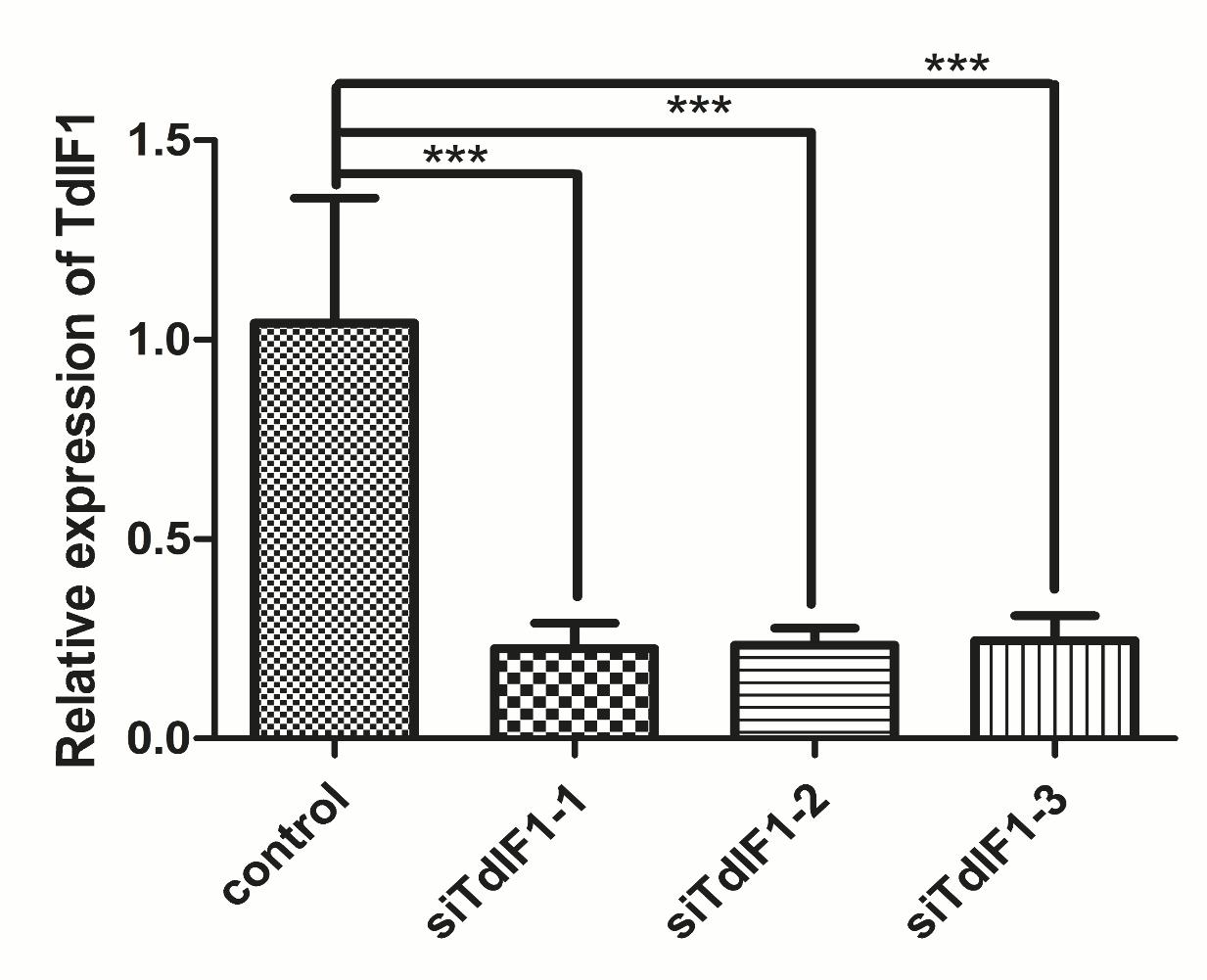


**Figure S1. Screening of different siRNAs for gene silencing of TdIF1.** The A549 cells were transfected with siTdIF1-1 (siRNA sequence: TTCTCCGAACGTGTCACGT), siTdIF1-2 (siRNA sequence: GGAACATAATGATAAAGCA), siTdIF1-3 (siRNA sequence: GAGGAAGAATCTACATCAA) and control nonspecific siRNA for 48 h. Relative expression of TdIF1 was analyzed by real-time RT-PCR. The expression of TdIF1 was normalized to GAPDH. Error bars represent the standard deviation of 3 experiments (***, P<0.001).
